# Low-Molecular-Weight Fucoidan Induces Endothelial Cell Migration via the PI3K/AKT Pathway and Modulates the Transcription of Genes Involved in Angiogenesis

**DOI:** 10.3390/md13127075

**Published:** 2015-12-18

**Authors:** Claire Bouvard, Isabelle Galy-Fauroux, Françoise Grelac, Wassila Carpentier, Anna Lokajczyk, Sophie Gandrille, Sylvia Colliec-Jouault, Anne-Marie Fischer, Dominique Helley

**Affiliations:** 1 Inserm, UMR-S765, 75006 Paris, France; francoise.grelac@parisdescartes.fr; 2 Université Paris Descartes, Sorbonne Paris Cité, 75006 Paris, France; isabelle.galy-fauroux@parisdescartes.fr (I.G-F.); anna.lokajczyk@parisdescartes.fr (A.L.); sophie.gandrille@parisdescartes.fr (S.G.); anne-marie.fischer@egp.aphp.fr (A-M.F); dominique.helley@egp.aphp.fr (D.H.); 3 Université Paris Diderot, Sorbonne Paris Cité, 75013 Paris, France; 4 Inserm, UMR-S970, 75015 Paris, France; 5 Plateforme P3S, Sorbonne Universités, 75013 Paris, France; wassila.carpentier@upmc.fr; 6 Inserm, UMR-S1140, 75006 Paris, France; 7 AP-HP, Hôpital Européen Georges Pompidou, 75015 Paris, France; 8 IFREMER, Route de l’Ile d’Yeu, 44300 Nantes, France; sylvia.colliec.jouault@ifremer.fr

**Keywords:** fucoidan, angiogenesis, vasculogenesis, migration, signaling, transcriptomics

## Abstract

Low-molecular-weight fucoidan (LMWF) is a sulfated polysaccharide extracted from brown seaweed that presents antithrombotic and pro-angiogenic properties. However, its mechanism of action is not well-characterized. Here, we studied the effects of LMWF on cell signaling and whole genome expression in human umbilical vein endothelial cells and endothelial colony forming cells. We observed that LMWF and vascular endothelial growth factor had synergistic effects on cell signaling, and more interestingly that LMWF by itself, in the absence of other growth factors, was able to trigger the activation of the PI3K/AKT pathway, which plays a crucial role in angiogenesis and vasculogenesis. We also observed that the effects of LMWF on cell migration were PI3K/AKT-dependent and that LMWF modulated the expression of genes involved at different levels of the neovessel formation process, such as cell migration and cytoskeleton organization, cell mobilization and homing. This provides a better understanding of LMWF’s mechanism of action and confirms that it could be an interesting therapeutic approach for vascular repair.

## 1. Introduction

Low-molecular-weight fucoidan (LMWF) is a sulfated polysaccharide extracted from the brown algae *Ascophyllum nodosum* that presents potential therapeutic properties in the prevention and treatment of atherosclerosis/thrombosis related diseases. Its structure and composition are comparable to low-molecular-weight heparin (LMWH), which is widely used in the clinic as an antithrombotic agent. In a rabbit model of arterial thrombosis, LMWF was more effective than LMWH to prevent venous and arterial thrombosis, with a reduced hemorrhagic risk [1,2]. It has also been demonstrated that a 8 kDa LMWF reduces vascular muscle cell proliferation and prevents neointimal hyperplasia in a rabbit in-stent restenosis model [3,4]. In a rat model of unilateral hindlimb ischemia, intramuscular injection of LMWF promotes post-ischemic reperfusion and increases capillary density and muscle regeneration [5]. LMWF has also been shown to enhance the effects of proangiogenic growth factors such as fibroblast growth factor 2 (FGF-2) and vascular endothelial growth factor (VEGF), both *in vitro* and *in vivo* [5,6,7], as it enhances the binding of VEGF to its receptors VEGFR2 and NRP1 [8]. Vascular remodeling occurs after vascular injury or tissue ischemia to repair/re-endothelialize the injured blood vessels or to create new ones. The formation of new blood vessels results from two distinct processes called angiogenesis and vasculogenesis. Neovessel formation is a multi-step process involving the secretion of cytokines and growth factors at the site of injury/ischemia, the degradation of the vessel wall by proteases, the migration and proliferation of endothelial cells that are already present *in situ* (angiogenesis), and also the mobilization of endothelial progenitors from the bone marrow and their recruitment at the site of neovessel formation (vasculogenesis). Re-endothelialization of the luminal surface of an injured vessel (for example after stent implantation) also involves mature endothelial cells and circulating endothelial progenitors [9].

Endothelial colony-forming cells (ECFCs), a subtype of endothelial progenitors characterized by the ability to form blood vessels *in vivo*, represent a potential autologous cell therapy product for vascular regeneration as they can be isolated from blood or bone marrow and expanded *in vitro*. In preclinical animal models, injection of ECFCs promotes neovessel formation and protects ischemic tissues from necrosis [10]. However, endothelial progenitor based cell therapy has shown limited efficacy in the clinical setting. It is thus crucial to find strategies to enhance ECFC proangiogenic potential.

We and others have previously demonstrated that LMWF has proangiogenic effects on both mature endothelial cells and ECFCs [6,7,11]. Stimulation of ECFCs with LMWF prior to their intravenous injection in mice with hindlimb ischemia increases the efficiency of the cell therapy, with a significant increase in foot perfusion and a significant reduction in ischemic muscle necrosis. *In vitro*, stimulation of ECFCs with LMWF increases adhesion to activated endothelium in flow conditions and promotes transendothelial migration and pseudotube formation. LMWF binds to the ECFC outer membrane, and is internalized within 15 min [11]. However, the mechanisms of action of LMWF on endothelial cells and their progenitors are not well characterized. Here we studied the effects of LMWF on several signaling pathways and on gene expression, using human umbilical vein endothelial cells (HUVECs) as a model of mature endothelial cells and ECFCs from human umbilical cord blood as a model of endothelial progenitors.

## 2. Results and Discussion

### 2.1. LMWF Enhances VEGF-Induced Activation of Signaling Pathways

**Figure 1:**
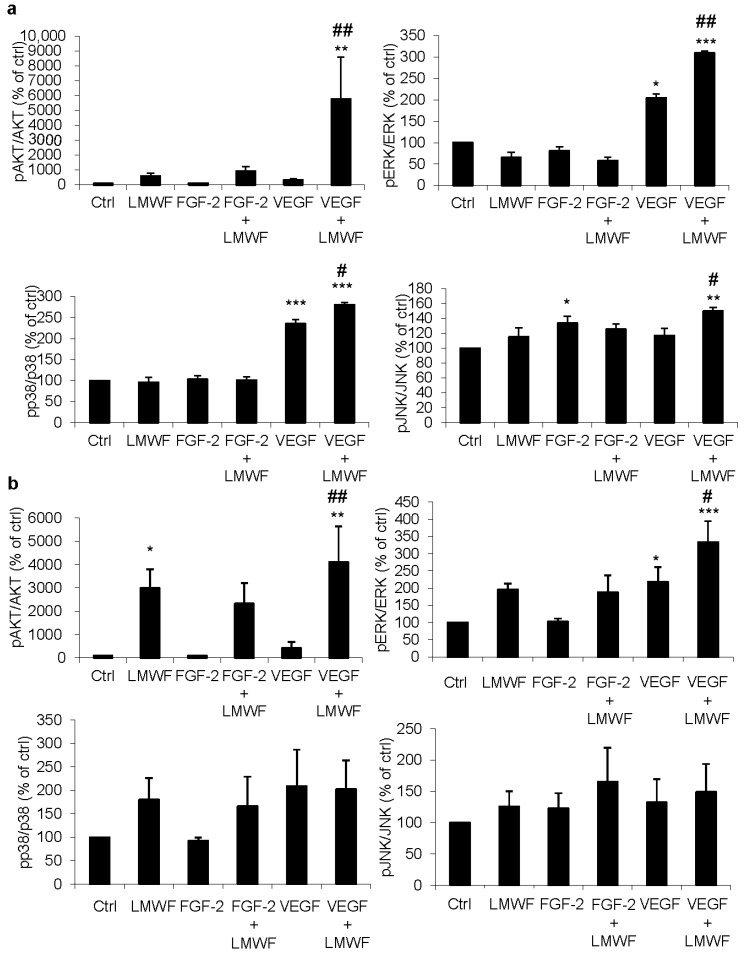
Bio-Plex analysis of signal transduction induced by LMWF, in association with FGF-2 and VEGF on ECFCs (**a**) and HUVECs (**b**). Phosphorylation of AKT, ERK, p38 and JNK in response to the different treatments: no treatment (ctrl); LMWF (10 μg/mL), FGF-2 (5 ng/mL); FGF-2 (5 ng/mL) and LMWF (10 μg/mL); VEGF (40 ng/mL); VEGF (40 ng/mL) and LMWF (10 μg/mL). Data are ratios of phospho-protein to the total protein, normalized to the untreated group (ctrl) and are represented as the mean + SEM of three independent experiments. * *p* < 0.05, ** *p* < 0.01, *** *p* < 0.001 *vs.* untreated group (ctrl). ^#^ *p* < 0.05, ^##^ *p* < 0.01 *vs.* VEGF treated group.

For ECFCs, LMWF significantly enhanced VEGF-induced phosphorylation of ERK and p38, and LMWF and VEGF seemed to have synergistic effects on the phosphorylation of AKT and JNK. Indeed, the association of LMWF and VEGF induced a significant increase in AKT, ERK, p38 and JNK phosphorylation, compared to the untreated group, to the VEGF treated group or to the LMWF treated group. (Figure 1a)

For HUVECs, no significant effects of the different treatments were observed on p38 and JNK phosphorylation, but LMWF significantly enhanced VEGF-induced ERK phosphorylation. Interestingly, although the association of VEGF and LMWF induced a strong and significant increase in AKT phosphorylation, compared to the untreated group or to the VEGF treated group, the difference was not statistically significant compared to the LMWF treated group since we observed that LMWF by itself induced a strong phosphorylation of AKT in HUVECs, with a ratio increased by 30 times compared to the untreated group (*p* < 0.05). (Figure 1b) A 7-fold increase in AKT phosphorylation was also observed in ECFCs treated with LMWF alone, compared to the untreated group, but this difference was not statistically significant due to a high variability (ECFCs from one cord blood in particular showed an exacerbated response when analyzed with Bioplex^®^). (Figure 1a)

For both ECFCs and HUVECs, the association of LMWF and FGF-2 did not significantly increase the activation of these signaling pathways compared to the untreated group or to the FGF-2 treated group. (Figure 1a,b).

### 2.2. LMWF but Not LMWH Induces AKT Phosphorylation in ECFCs and HUVECs

**Figure 2:**
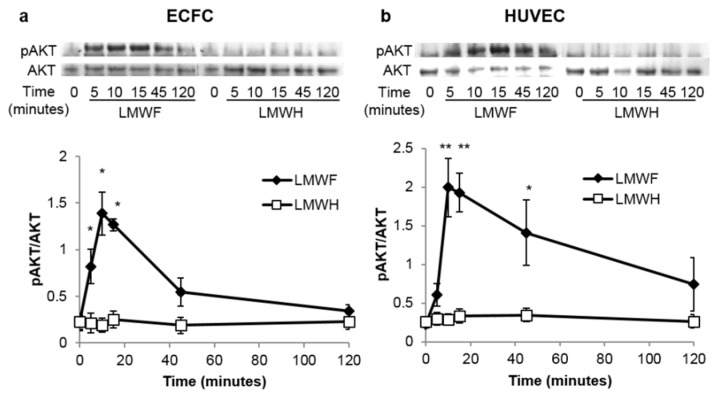
LMWF but not LMWH induces the phosphorylation of AKT in ECFCs (**a**) and HUVECs (**b**) in a time dependent manner. Cells were treated with LMWF or with LMWH (10 μg/mL) for 5, 10, 15, 45 and 120 min and then washed and lysed. Phosphorylated AKT and total AKT were quantified by Western blot analysis. Data are represented as a ratio of pAKT to AKT and are the mean ± SEM of at least 3 independent experiments. * *p* < 0.05, ** *p* < 0.01 *vs.* LMWH treated group.

AKT phosphorylation is rapidly induced by LMWF in ECFCs and in HUVECs, the pAKT/AKT ratio reaches its maximal level after 10–15 min of stimulation (*p* < 0.05 *vs.* LMWH for ECFCs and *p* < 0.01 *vs.* LMWH for HUVECs), and then progressively returns to the basal level. This activation of the PI3K/AKT pathway seems specific to LMWF, as LMWH did not induced any phosphorylation of AKT. (Figure 2a,b)

### 2.3. LMWF Enhances ECFC and HUVEC Cell Migration in a PI3K-Dependent Manner

**Figure 3:**
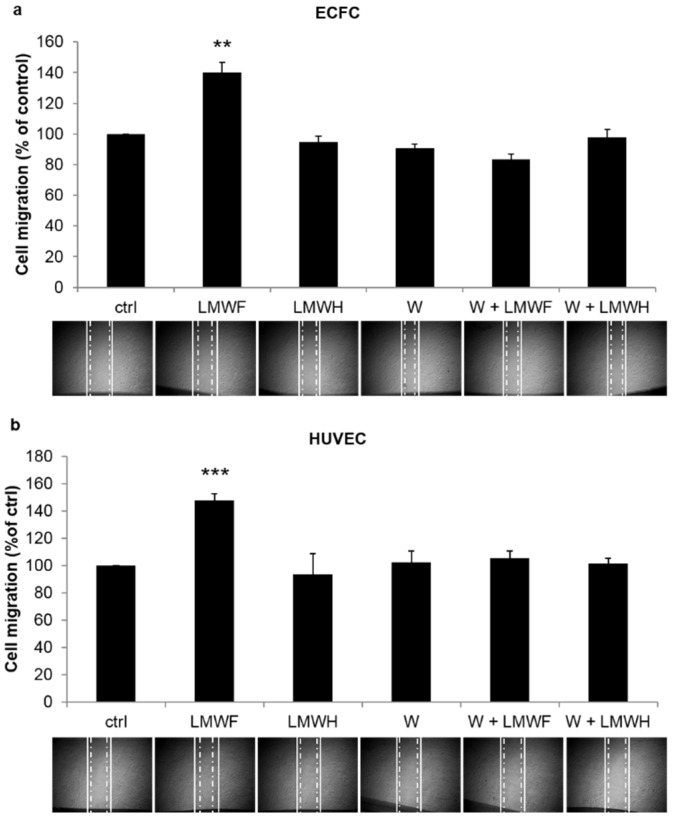
LMWF but not LMWH induces cell migration in a PI3K-dependent manner. ECFC (**a**) or HUVEC (**b**) monolayers were mechanically scratched with a sterile plastic pipette tip after a 24 h incubation with the following treatments: no treatment (ctrl); LMWF (10 μg/mL); LMWH (10 μg/mL); wortmannin (W, 100 nM); wortmannin (W, 100 nM) and LMWF (10 μg/mL); wortmannin (W, 100 nM) and LMWH (10 μg/mL). Microphotographs were taken just after the scratch and 6 h later to quantify the surface covered by migrating cells (between the solid and the dash-dot lines). Data are normalized to the untreated group and are the mean + SEM of 3 independent experiments. ** *p* < 0.01, *** *p* < 0.001 *vs.* all other groups.

Pretreatment with LMWH had no significant effect on ECFC or HUVEC migration, whereas pretreatment with LMWF enhanced the migration of ECFCs by 40% (*p* < 0.01) and of HUVECs by 48% (*p* < 0.001) compared to the untreated group. When PI3K activation was inhibited by wortmannin, the effect induced by LMWF on cell migration was lost, with no significant difference with the untreated group. (Figure 3a,b)

### 2.4. Transcriptomic Analysis Reveals that LMWF Modulates the Expression of Genes Involved in Angiogenesis, Vasculogenesis and Cell Migration

**Figure 4:**
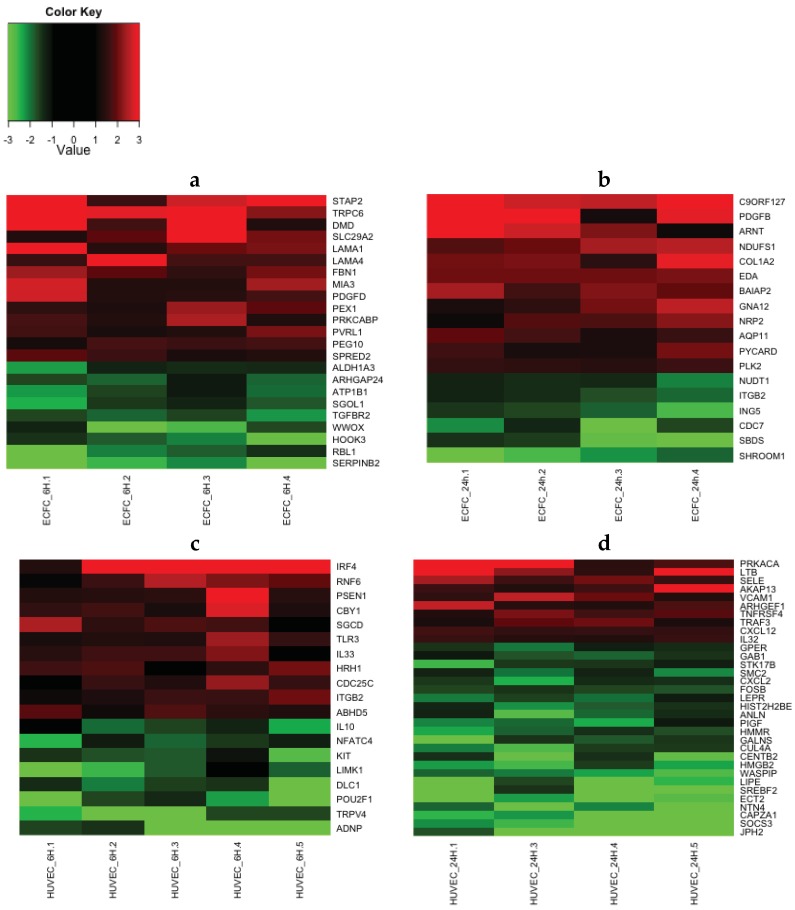
Gene expression heatmaps generated using illumina beadchips data. ECFCs (**a**,**b**) and HUVECs (**c**,**d**) were treated with 10 μg/mL of LMWF for 6 h (**a**,**c**) or 24 h (**b**,**d**). RNA was extracted and analyzed using Illumina Human HT-12 Expression beadchips. The heatmaps show the expression fold change of a selection of differentially expressed genes, with an expression fold change greater than 1.5 fold and a significance *p*-value < 0.05. An up-regulation will appear red and a down-regulation will appear green. At least four biologically independent replicates are represented.

**Figure 5:**
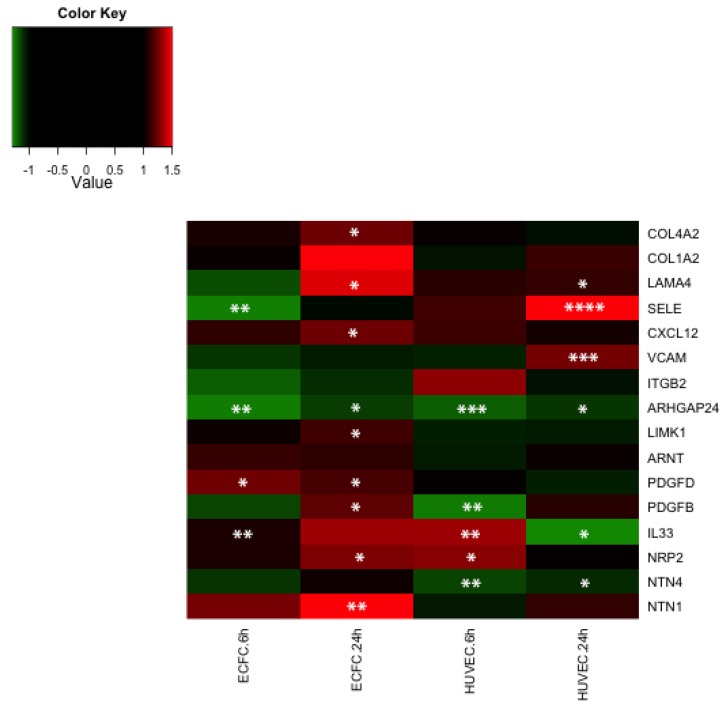
Gene expression heatmap generated using qRT-PCR data. ECFCs and HUVECs were treated with 10 μg/mL of LMWF for 6 h or 24 h. RNA was extracted, cDNA was synthesized and analyzed using Taqman technology. mRNA levels were measured by quantitative RT-PCR using total RNA isolated from ECFC or HUVEC. The heatmap shows for each gene the mean of the expression fold change obtained from at least four biologically independents replicates. An up-regulation will appear red and a down-regulation will appear green. * *p* < 0.05, ** *p* < 0.01, *** *p* < 0.001, **** *p* < 0.0001 *vs.* untreated control.

LMWF significantly enhanced or decreased the expression of 116 to 196 genes (depending on the cell type and time point), with an expression fold change greater than 1.5 and a *p*-value < 0.05 (Table 1). Using the Ingenuity^®^ interface we performed a functional analysis of these lists of significantly differentially expressed genes. Some functions were modulated in both cell types (ECFC and HUVEC) and both time points (6 h and 24 h), such as cell movement, cellular assembly and organization, cell morphology, cellular growth and proliferation, cardiovascular system development and function, hematological system development and function, hematopoiesis, immune cell trafficking, inflammatory response, and cell-to-cell signaling and interaction (Table 2). In particular, LMWF modulated the expression of genes involved in the formation and branching of endothelial tubes (*ARHGAP24*, *TGFBR2*, *LAMA4*), in wound healing (*SERPINB2*, *MIA3*), in leukocyte mobilization and rolling (*CXCL12*, *SELE*, *VCAM1*, *SOCS3*), in oriented migration and/or cytoskeletal organization (*LIMK1*, *ARHGAP24*, *BAIAP2*, *TGFBR2*, *GNA12*, *SHROOM1*) and axon guidance (*NRP2*, *NTN1*, *NTN4*). LMWF modulated the expression of genes coding for extracellular matrix proteins (*LAMA1*, *LAMA4*, *COL1A2*, *COL4A2*), growth factors or cytokines (*IL 10*, *IL 33*, *CXCL12*, *PDGFB*, *PDGFD*), adhesion molecules (*SELE*, *VCAM1*), growth factors (*ARNT*) and intracellular proteins (*LIMK1*, *BAIAP2*, *ECT2*, *GNA12*) involved in either angiogenesis, vasculogenesis or cell migration. We generated heatmaps to represent the expression fold change of selected genes involved in the relevant functions discussed above (Figure 4), and 16 genes were selected for further quantitative real-time PCR confirmation (Figure 5). Microarray data are available in the ArrayExpress database (www.ebi.ac.uk/arrayexpress) under accession number E-MTAB-3925.

**Table 1:** Number of genes that were differentially expressed upon treatment with 10 μg/mL LMWF. ECFCs and HUVECs were treated with 10 μg/mL of LMWF for 6 h or 24 h. RNA was extracted and analyzed using Illumina Human HT-12 Expression beadchips. After filtering probes with detection *p*-values < 0.05 for at least half of the arrays, differentially expressed genes with an expression fold change greater than 1.5 fold were selected using the Student test, with a significance *p*-value < 0.05, including Bonferroni and Benjamini Hochberg false discovery detection. Each dataset was derived from at least four biologically independent replicates.

Number of Genes	ECFC 6 h	ECFC 24 h	HUVEC 6 h	HUVEC 24 h
upregulated	110	66	74	65
downregulated	41	50	91	128
Total	151	116	165	193

**Table 2:** Functional analysis of differentially expressed genes upon treatment with 10 μg/mL LMWF. ECFCs and HUVECs were treated with 10 μg/mL of LMWF for 6 h or 24 h. RNA was analyzed using Illumina Human HT-12 Expression beadchips. Differentially expressed genes with an expression fold change greater than 1.5 fold were selected using the Student test, with a significance *p*-value < 0.05. Each dataset was derived from at least four biologically independent replicate samples. The generated lists were interpreted using the Ingenuity^®^ interface. A selection of the most relevant functions that were modulated in most conditions is represented below, along with the number of differentially expressed genes (*n*) for each function, and the corresponding *p*-value range.

Number of Genes (*n*) by Function	ECFC 6 h		ECFC 24 h		HUVEC 6 h		HUVEC 24 h	
	*n*	*p*-value	*n*	*p*-value	*n*	*p*-value	*n*	*p*-value
Cardiovascular System Development and Function	5	4.64 × 10^−3^–1.38 × 10^−2^	5	5.73 × 10^−4^–3.24 × 10^−2^	4	5.78 × 10^−3^–3.98 × 10^−2^	11	2.93 × 10^−4^–4.84 × 10^−2^
Cell Morphology	4	4.64 × 10^−3^–2.3 × 10^−2^	6	3.29 × 10^−3^–4.51 × 10^−2^	11	1.58 × 10^−3^–3.98 × 10^−2^	14	1.33 × 10^−3^–4.84 × 10^−2^
Cell-To-Cell Signaling and Interaction	7	4.64 × 10^−3^–3.65 × 10^−2^	8	3.03 × 10^−3^–4.83 × 10^−2^	10	9.03 × 10^−4^–4.99 × 10^−2^	19	1.47 × 10^−4^–4.84 × 10^−2^
Cellular Assembly and Organization	8	4.64 × 10^−3^–4.98 × 10^−2^	10	1.4 × 10^−3^–4.76 × 10^−2^	7	1.58 × 10^−3^–3.98 × 10^−2^	15	4.93 × 10^−5^–4.84 × 10^−2^
Cellular Growth and Proliferation	4	4.64 × 10^−3^–4.1 × 10^−2^	4	3.29 × 10^−3^–4.51 × 10^−2^	8	3.3 × 10^−3^–4.53 × 10^−2^	30	1.82 × 10^−3^–4.51 × 10^−2^
Cellular Movement	2	1.84 × 10^−2^–2.75 × 10^−2^	6	4.7 × 10^−4^–4.83 × 10^−2^	7	4.88 × 10^−4^–4.53 × 10^−2^	15	1.38 × 10^−5^–4.84 × 10^−2^
Hematological System Development and Function	4	4.64 × 10^−3^–4.1 × 10^−2^	4	3.29 × 10^−3^–4.83 × 10^−2^	10	4.88 × 10^−4^–4.99 × 10^−2^	15	4.93 × 10^−5^–4.84 × 10^−2^
Hematopoiesis	2	4.64 × 10^−3^–4.1 × 10^−2^	2	9.84 × 10^−3^–3.88 × 10^−2^	7	4.88 × 10^−4^–4.57 × 10^−2^	7	1.46 × 10^−4^–4.84 × 10^−2^
Immune Cell Trafficking	0	n/a	3	3.29 × 10^−3^–4.83 × 10^−2^	7	4.88 × 10^−4^–4.99 × 10^−2^	7	4.93 × 10^−5^–4.84 × 10^−2^
Inflammatory Response	2	1.84 × 10^−2^–4.1 × 10^−2^	4	3.29 × 10^−3^–4.83 × 10^−2^	8	4.88 × 10^−4^–4.57 × 10^−2^	8	4.93 × 10^−5^–4.55 × 10^−2^

### 2.5. Discussion

It has been previously reported that LMWF potentiates the proangiogenic effects of growth factors such as FGF2 or VEGF [5,6,7,8,12]. Here we observed that LMWF enhances the intensity of the activation of several intracellular signaling pathways triggered by growth factors such as VEGF or FGF2 in mature endothelial cells (HUVECs) and endothelial progenitors (ECFCs), which could be explained by the fact that LMWF enhances the binding of VEGF to its receptors, as previously reported [8]. More interestingly, we observed that LMWF could induce the phosphorylation of AKT, without the presence of VEGF or FGF2, in both HUVECs and ECFCs. This activation of the PI3K/AKT pathway seemed specific to LMWF, as another sulfated polysaccharide LMWH was unable to induce AKT phosphorylation. These results suggest that LMWF has specific and inherent VEGF and FGF2-independent properties and mechanisms, which we decided to further investigate at a cellular and transcriptional level.

The PI3K/AKT pathway plays a crucial role in angiogenesis and vasculogenesis. It regulates endothelial cell survival, actin reorganization and migration [13], capillary-like formation, vessel integrity and cardiovascular homeostasis by phosphorylating multiple angiogenic substrates including eNOS and Fork-head box proteins [14,15]. Treatments that increase ECFC proangiogenic properties and cell therapy efficiency in ischemic mice also seem to increase AKT phosphorylation *in vitro* [10,11,16]. The PI3K/AKT pathway has also been shown to induce early endothelial progenitor proliferation and differentiation *in vivo* [17]. In mouse hindlimb ischemia models, AKT plays a role in the mobilization and recruitment of early endothelial progenitors [18], and genetic deletion of Pi3Kγ in mice impairs post-ischemic neovascularization and early endothelial progenitors functions [19].

Interestingly, cellular movement and cellular assembly and organization (including migration, remodeling of cytoskeleton, chemotaxis, mobilization, rolling, adhesion) were the main functions regulated at a transcriptional level by LMWF after 24 h of treatment for both HUVECs and ECFCs. In keeping with these results we observed that LMWF increased cell migration in a PI3K/AKT dependent manner since the effects of LMWF on both ECFC and HUVEC migration were lost when the activation of the PI3K/AKT pathway was inhibited by wortmannin.

LMWF modulated the expression of genes directly involved in cytoskeletal organization such as *GNA12* (also known as *ARHGEF1*), *SHROOM1*, *LIMK1* and *ARHGAP24*. The expression of *ARHGAP24*, coding for the Rho GTPase activating protein 24 (RhoGAP24), was downregulated upon LMWF treatment at 6 and 24 h in both HUVECs and ECFCs. RhoGAP24 modulates the activation of Rac, cdc42 and Rho and therefore mediates cytoskeletal organization, cell morphology, cell polarity and cell motility. In fibroblasts or melanoma cells, RhoGAP24 inactivates Rac and consequently negatively regulates the formation of actin stress fibers, lamellipodia and filopodia, necessary for cell motility [20,21] and a vascular-specific and Rho-specific isoform has been shown to modulate endothelial cell migration and tube formation *in vitro* and *in vivo* [22]. *LIMK1* expression was upregulated in ECFCs at 24 h. LIMK1 acts downstream of Rho family GTPases and phosphorylates cofilin, which inhibits its actin-depolymerization activity. *LIMK1* overexpression has been shown to increase cell migration capability [23]. Interestingly, relationships between PI3K or AKT and Rho family GTPases have been demonstrated in several studies. For example phosphorylated AKT colocalizes with Rac and cdc42 at the leading edge of fibroblasts [24] and multiple Rho family GTPases are involved in a positive feeback loop that activates PI3K [25].

Vasculogenesis involves ECFC mobilization from the bone marrow, chemoattraction, rolling and adhesion to activated endothelium. LMWF modified the expression of genes involved in mobilization and homing (most significantly modified functions for HUVECs at 24 h, with *p*-values of 1.38E-5 and 1.47E-4, respectively) such as *CXCL2*, *CXCL12*, *SELE*, *SOCS3*, *VCAM1*. LMWF upregulated the expression of the chemokine CXCL12 (also known as SDF-1). CXCL12 plays a key role in the mobilization and homing of leukocytes, by activating its receptor CXCR4. CXCR4 is also expressed at the surface of ECFCs, and CXCL12 has been shown to increase their adhesion, migration, survival, tube formation, as well as their integration in immature vascular networks *in vitro* [26,27,28]. In addition, in hindlimb ischemia mouse models, local injection of CXCL12 or injection of ECFCs pretreated with CXCL12 promotes the recruitment of ECFCs and the reperfusion of the ischemic limb [27,28]. Interestingly, intravenous injection of LMWF increases CXCL12 serum levels in rats [5]. LMWF downregulated *SOCS3* expression in HUVECs. SOCS3 negatively regulates the mobilization of hematopoietic progenitors induced by cytokines such as G-CSF or CXCL12 [29].

LMWF increased the expression of *SELE* and *VCAM1* at 24 h. E-selectin and VCAM1 are adhesion molecules that are expressed at the surface of activated endothelial cells and mediate leukocyte and ECFC rolling and adhesion along the vessel wall [27]. E-selectin plays a pivotal role in ECFC homing to ischemic limb and angiogenesis [30]. CXCL12 increases the expression of E-selectin ligands at the surface of ECFCs and further facilitates their adhesion [31]. The soluble form of E-selectin stimulates endothelial cell migration and neovessel formation *in vivo* [32].

LMWF increased the expression of genes coding for the proangiogenic growth factors PDGFB and PDGFD by ECFCs. PDGFB is a potent mitogen for cells of mesenchymal origin that mediates blood vessel development and wound healing. PDGFB is secreted by endothelial cells and promotes the proliferation and recruitment of pericytes and vascular smooth muscle cells [33]. It has recently been shown that ECFCs support mesenchymal stem sells (MSC) engraftment and regenerative capacity *in vivo* via PDGFB secretion [34]. In addition our group demonstrated that LMWF increases MSC differentiation potential [35]. Regenerative therapy strategies combining LMWF, ECFCs and MSCs could thus be an interesting approach. PDGFD has been identified more recently and also promotes wound healing, by increasing macrophage recruitment and blood vessel maturation [36].

Genes coding for extracellular matrix proteins such as laminin and collagen were also upregulated by LMWF. Notably, the gene *LAMA4*, coding for the laminin α4 chain, a subunit of laminin 411, which is the major component of the endothelial basement membrane and plays a crucial role in vessel maturation [37] and branching [38], was upregulated in both HUVECs and ECFCs.

LMWF also modulated the expression of members of the neuropilin and netrin families. These secreted molecules are well known axonal guidance cues, and their role in angiogenesis and especially in vascular patterning has been discovered more recently [39]. *NRP2* was upregulated in both HUVECs and ECFCs. Neuropilin 2 acts as a co-receptor for VEGF receptors in response to VEGF-A and VEGF-C. The interaction of neuropilin 2 with VEGFR2 enhances VEGFR2 phosphorylation and eventually promotes endothelial cell survival and migration [40]. LMWF induced an upregulation of *NTN1*, while *NTN4* seemed to be downregulated. It is not clear yet whether netrin-1 and netrin-4 are pro- or anti-angiogenic factors. For example, Wilson *et al.* [41] showed that netrin-1 and netrin-4 enhance endothelial cell migration, proliferation, pseudotube formation and post-ischemic angiogenesis *in vivo*, whereas Larrivee *et al.* [42] and Lejmi *et al.* [43] observed antiangiogenic effects. The effects of these factors might depend on several parameters such as the level of expression of their different receptors, the type of endothelial cell and their state of activation.

Since the genes that we found to be up- or downregulated by LMWF were associated with functions that are known to be regulated by the PI3K/AKT pathway (such as migration, remodeling of cytoskeleton, chemotaxis, mobilization, rolling and adhesion), it is possible that the activation of this pathway by LMWF subsequently leads (directly or indirectly) to the modulation of the expression of these genes. Unfortunately, a very limited amount of data is available regarding the effects of PI3K/AKT on gene expression in endothelial cells (most studies focus on functional assays, and occasionally investigate a small number of genes, but not whole genome expression). So far, among the genes we selected, only the expression of *PDGFB* and *VCAM1* has been shown to be modulated via the PI3K/AKT pathway in endothelial cells under specific conditions [44,45], to our knowledge. Several downstream effectors of PI3K/AKT have been identified in endothelial cells, such as endothelial nitric oxide synthase (eNOS), which generates NO through the NADPH-dependent oxidation of L-arginine, forkhead transcription factors (such as Foxo1, Foxo3a and Foxo4), and RhoA (for a specific PI3K isoform p110alpha) [46]. Most of the genes that we found to be up- or downregulated by LMWF have not been reported as Foxo1 or Foxo3A target genes in endothelial cells, *PDGFB* being the exception [47], and to our knowledge no data is available for these other specific downstream effectors. It is thus likely that LMWF acts through other downstream effectors than Foxo1 and Foxo3A, and additional mechanisms (that may also be PI3K/AKT independent or that may involve this pathway only indirectly) may be involved and remain to be further studied. In addition, in endothelial cells PI3K is activated downstream of various receptors such as G protein coupled receptors (such as CXCR4), tyrosine kinase receptors (such as VEGF receptors), integrins, and death receptors (such as tumor necrosis factor α receptor), or it can also result from a lack of inactivation by PTEN (negative regulator of PI3K signaling) [46]. It would be interesting to investigate in the future if LMWF activates the PI3K/AKT pathway via one of these upstream receptors, via PTEN or via a novel effector, since the downstream signaling cascade and subsequent potential effects on gene expression will likely depend on which upstream effectors are involved.

It is also important to note that the fucoidan used in this study is a 4.5 kDa LMWF extracted from the brown algae *Ascophyllum nodosum*. This fraction has been studied in several publications from our group for its pro-angiogenic [6,7,11] and anti-thrombotic [2] properties. Our data provide new insight into the effects and mode of action of this particular LMWF fraction, and support its potential benefits for vascular repair, in accordance with these previous studies. However, the conclusions from this study cannot be extrapolated to other fucoidans that may have been extracted from other algae species and may present different molecular weight and sulfate content, since these parameters can affect biological activity [48].

## 3. Experimental Section

### 3.1. Reagents

LMWF was obtained from the brown algae *Ascophyllum nodosum* using a radical depolymerization process adapted from Nardella *et al.* [49], as previously described [2,6,7,11,50]. The average molecular weight of the fraction used is 4500 Da (polydispersity 1.7), the fucose content is 35% (w/w), the uronic acid content is 3% (w/w) and the sulfate content is 34% (w/w). We used the LMWH enoxaparin from Sanofi-Aventis. Its average molecular weight is 4500 Da (polydispersity 1.3), and its sulfate content is 40% (w/w), which is very close to the LMWF fraction that we are using in this study. The structures of LMWF and LMWH are represented in [35]. Recombinant human VEGFA-165 was purchased from Abcys (Paris, France) recombinant human FGF-2 from R&D systems (Abingdon, UK) and wortmanin from Calbiochem (Billerica, MA, USA)

### 3.2. ECFC Isolation and Culture

Mononuclear cells were isolated from human umbilical cord blood by density gradient centrifugation as previously described [10]. After an adhesion step, CD34+ cells were selected by magnetic activated cell sorting. The cells thus collected were plated on 0.2% gelatin-coated 24-well plastic culture dishes at a density of 5 × 10^5^ cells/well in EGM-2 medium (Lonza, Walkersville, MD, USA). After 4 days, non adherent cells were removed and the medium was renewed. After 10–20 days of culture, ECFC colonies became visible microscopically. Cells were then detached with trypsin and expanded in EGM-2 on 0.2% gelatin coated plates and grown at 37 °C in a humidified 5% CO_2_ atmosphere for further analysis. ECFC were used 25 to 45 days after cord blood processing. ECFCs were positive for CD31, CD34, CD144 and CD146 but not for the monocytic markers CD45 and CD14. (For complete ECFC characterization, see [10]).

### 3.3. HUVEC Isolation and Culture

Endothelial cells from the vein of human umbilical cord were isolated by enzymatic digestion with collagenase as previously described [7] and cultured on 0.2% gelatin coated plates, in EGM-2 medium in a humidified 5% CO_2_ atmosphere at 37 °C. HUVECs were used at passage 2 or 3.

### 3.4. Bio-Plex^®^ Analysis

ECFCs and HUVECs were seeded in 6-well 0.2% gelatin coated plates at a density of 2 × 10^5^ cells/well. Two days later, when cells reached subconfluence they were placed overnight in EBM-2 + 5% FCS. The next morning, cells were washed with PBS and serum starved for 4 h in EBM-2.Cells were then stimulated during 15 min with FGF2 (5 ng/mL) or VEGFA-165 (40 ng/mL), in association with LMWF (10 μg/mL) or not. Cells were then washed and lysed using the Bio-Plex^®^ cell lysis kit (Biorad, Hercules, CA, USA). We used the BCA protein assay to ensure equal amounts of protein in all the lysates. Lysates were incubated with microbeads from Biorad coupled with antibodies specific to the following proteins: phospho-AKT (171V210752), phospho-ERK1/2 (171V222382), phospho-p38 (171V213362), phospho-JNK (171V210342), total AKT (171V310752), total ERK1/2 (171V322382), total p38 (171V313362), total JNK (171V310342), processed according to the manufacturer’s protocol, and analyzed using a Bio-plex^®^ suspension array system. Results are expressed as the ratio of the phospho-protein to the total protein, normalized to the untreated group and represented as means + SEM of three independent experiments.

### 3.5. Western Blot Analysis

ECFCs and HUVECs were seeded in 6-well 0.2% gelatin coated plates at a density of 2 × 10^5^ cells/well. Two days later, when cells reached subconfluence, they were placed overnight in EBM-2 + 5% FCS. The next morning, cells were washed with PBS and serum starved for 4 h in EBM-2. Cells were then stimulated with 10 μg/mL of LMWF or LMWH. After 5, 10, 15, 45 or 120 min of stimulation, wells were washed with PBS and cells were lysed in NuPAGE lysis buffer. Equal amounts of protein from each group were resolved by SDS/Page on a 8% acrylamide gel and probed by immunoblotting using anti-pAKT and anti-AKT antibodies (Cell signaling, Danvers, MA, USA). Densitometric readings were obtained using ImageJ software. Results are expressed as the ratio of pAKT to total AKT densitometric readings and represented as means ± SEM of at least three independent experiments.

### 3.6. In Vitro Wound Healing Assay

ECFCs and HUVECs were seeded in 6-well 0.2% gelatin coated plates at a density of 1 × 10^5^ cells/well. Three days later, cells were placed overnight in EBM-2 + 5% FCS. Cells were then treated with wortmanin 100 nM (Calbiochem) and with 10 μg/mL of LMWF or LMWH in EBM-2 + 5% FCS for 24 h (0.1% DMSO was present in all treatment conditions). A wound was created in the center of each well, by scratching the cell monolayer with a sterile plastic pipette tip. Cellular debris were removed as the wells were washed with PBS and the medium replaced by EBM-2 + 5% FCS. The wells were observed and imaged using a phased microscope just after the creation of the wound and 6 h later. The uncovered surface was measured by a blinded observer using Histolab software (Microvisions, Evry, France). Results are expressed as a surface covered by migrating cells, (difference between the uncovered surface at *t* = 0 and the uncovered surface at *t* = 6 h), normalized to the untreated group and represented as means + SEM of at least three independent experiments.

### 3.7. Transcriptome Analysis by Microarray

ECFCs and HUVECs were washed and placed overnight in EBM-2 + 2% FCS. The next morning, medium was replaced by EBM-2 + 2% FCS + 10 μg/mL LMWF or by EBM-2 + 2% FCS alone. After 6 h or 24 h of treatment, cells were washed with PBS, detached with accutase. After washing and centrifugation steps, the pellet was resuspended in RLT buffer (Qiagen, Venlo, The Netherlands) containing beta mercaptoethanol (Sigma, St Louis, MO, USA) and stored at −80 °C. Total RNA was isolated by using the RNeasy Mini Kit (Qiagen) as recommended by the manufacturer. Total RNA concentration and integrity was assessed by Nanodrop (Thermo Fisher Scientific, Waltham, MA, USA) and by microchip electrophoresis (Agilent technologies, Santa Clara, CA, USA). Total RNA was amplified using a kit from Ambion (Carlsbad, CA, USA) and applied to Human HT-12 expression beadchips (Illumina, San Diego, CA, USA), a genome wide array with 37,804 probes, according to the manufacturer’s instructions. Data were extracted and normalized using GeneSpring GX (Per Chip: Normalize to 50th percentile; Per Gene: Normalize to median). The working lists were created, filtering probes with detection *p*-values < 0.05 for at least half of the arrays. To control for systematic bias, unsupervised hierarchical clustering was performed, showing that samples are segregated according to the treatment groups. Differentially expressed genes with an expression fold change greater than 1.5 fold were selected using the Student test, with a significance *p*-value < 0.05, including Bonferroni and Benjamini Hochberg false discovery detection. Differential expression of the chosen genes was assessed using supervised hierarchical clustering. The interpretation of the resulting gene lists was performed using the Ingenuity^®^ interface. Heatmaps were generated with R. Each dataset was derived from at least four biologically independent replicate samples.

### 3.8. Quantitative Real-Time Reverse Transcription Polymerase Chain Reaction (qRT-PCR)

Total RNA was extracted using the RNeasy Mini Kit (Qiagen), as recommended by the manufacturer. cDNA synthesis was performed with the Reverse Transcriptase Core Kit (Eurogentec, Liege, Belgium). Gene expression were quantitatively analyzed by means of TaqMan Gene Expression assays (Applied Biosystems, Carlsbad, CA, USA) run on an ABI Prism 7900 HT Fast Real-Time PCR system (Applied Biosystems). The relative quantification method employed was based on the following arithmetic formula: −ΔΔCT, where ΔΔCT is the normalized signal level in a sample relative to the normalized signal level in the corresponding calibrator sample. The GAPDH gene was the endogenous control detector employed for normalization. Results are expressed as a fold change in gene expression compared to the untreated control.

### 3.9. Statistical Analysis

Bioplex, Western Blot, *in vitro* wound healing assay and qRT-PCR data were analyzed by ANOVA, followed by the Fisher protected least significant difference post hoc test, and implemented with STATVIEW software. Differences were assumed to be significant at *p* < 0.05.

## 4. Conclusions

In summary, LMWF modulates the expression of genes that are crucial for cell migration, angiogenesis and vasculogenesis. It phosphorylates AKT and induces the migration of mature or progenitor endothelial cells in a PI3K/AKT dependent manner. This provides a better understanding of the mechanism of action and effects of LMWF and supports its potential for therapeutic approaches in vascular repair such as vessel re-endothelialization or post-ischemic neovessel formation.
